# A unique case of paraneoplastic lymphomatoid Papuloerythroderma of Ofuji with atypical clinical features

**DOI:** 10.1016/j.jdcr.2024.02.009

**Published:** 2024-02-20

**Authors:** Oluwaseyi Adeuyan, Megan H. Trager, Emily R. Gordon, Brigit A. Lapolla, Celine M. Schreidah, Lauren M. Fahmy, Caroline Chen, Cynthia M. Magro, Larisa J. Geskin

**Affiliations:** a Columbia University Vagelos College of Physicians and Surgeons, New York, New York; b Department of Dermatology, Columbia University Irving Medical Center, New York, New York; c Division of Dermatopathology, Department of Pathology and Laboratory Medicine, Weill Cornell Medicine, New York, New York

**Keywords:** cutaneous T-cell lymphoma, dupilumab, hypereosinophilic syndrome, lymphoid reaction, Papuloerythroderma of Ofuji, paraneoplastic

## Introduction

Papuloerythroderma of Ofuji (PEO) is a rare dermatosis which presents as confluent pruritic papules in a background of erythroderma. There is characteristic sparing of the face and skin folds, referred to as the “deck-chair” sign.^1^ Histologically, an eczematous dermatitis is often seen.^2^ However, there are cases of PEO associated with mycosis fungoides (MF) and Sezary syndrome (SS) with significant lymphoid atypia on biopsy.^1^^,^^3^ Around 40% of PEO cases are associated with underlying disease, such as malignant lymphoma, infection (eg, HIV/AIDS, hepatitis C), and visceral malignancy (eg, gastric and colon cancers).^3^^,^^4^ We present the case of a 78-year-old man with erythroderma with histologic findings of erythrodermic T-cell dyscrasia found to have metastatic prostate adenocarcinoma. Treatment of the malignancy led to improvement in erythroderma. We propose that the patient had a reversible paraneoplastic lymphomatoid expression of PEO.

## Case report

A 78-year-old man with no prior dermatologic history presented to dermatology at Columbia University Medical Center (CUMC) with almost 1 year of pruritic “red dots” on the trunk. When the patient’s symptoms began, initial patch testing by an outside dermatologist was inconclusive. One month after the onset of the patient’s symptoms, the erythematous macules had progressed to widespread patches, and he endorsed fatigue, chills, and weight loss. The patient’s symptoms continued to have a waxing and waning course, notably flaring during an episode of COVID-19 8 months after his symptoms began, which temporarily responded to high-dose prednisone. However, over the course of the prednisone taper, the patient’s macules and patches returned. His skin and pruritus continued to worsen over the next 4 months, prompting his referral to CUMC.

**Fig 1:**
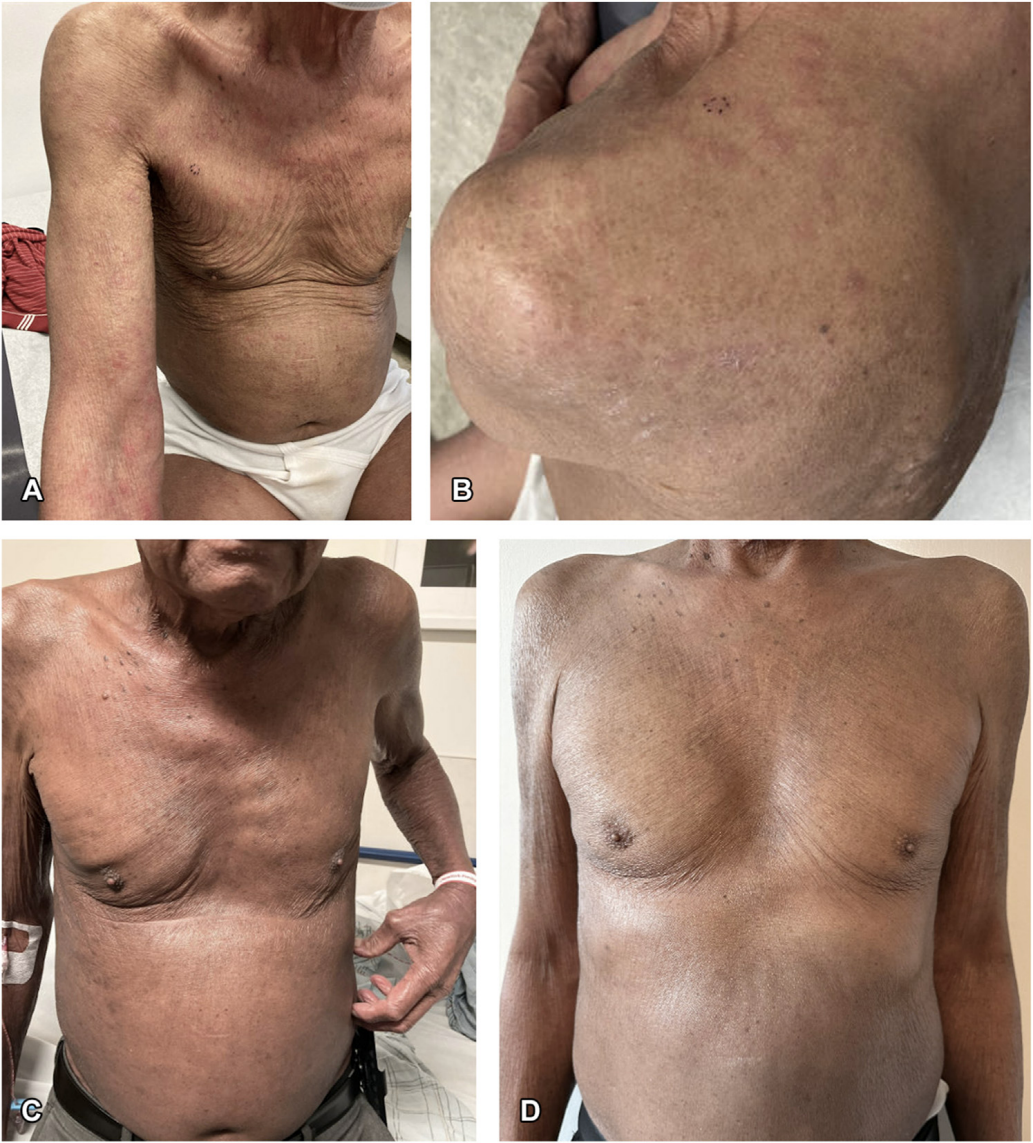
Clinical presentation. **A,** Erythematous macules and papules on the face, torso, skin folds, and extremities at initial visit. **B,** A closer view of the patient’s papules at initial presentation is shown. **C,** Diffuse scaling and thickened erythroderma, including skin folds, 4 months after the initial visit, consistent with disease progression. **D,** Improvement of the patient’s erythroderma after starting treatment with androgen deprivation therapy and an androgen receptor signaling inhibitor for his prostate cancer.

**Fig 2:**
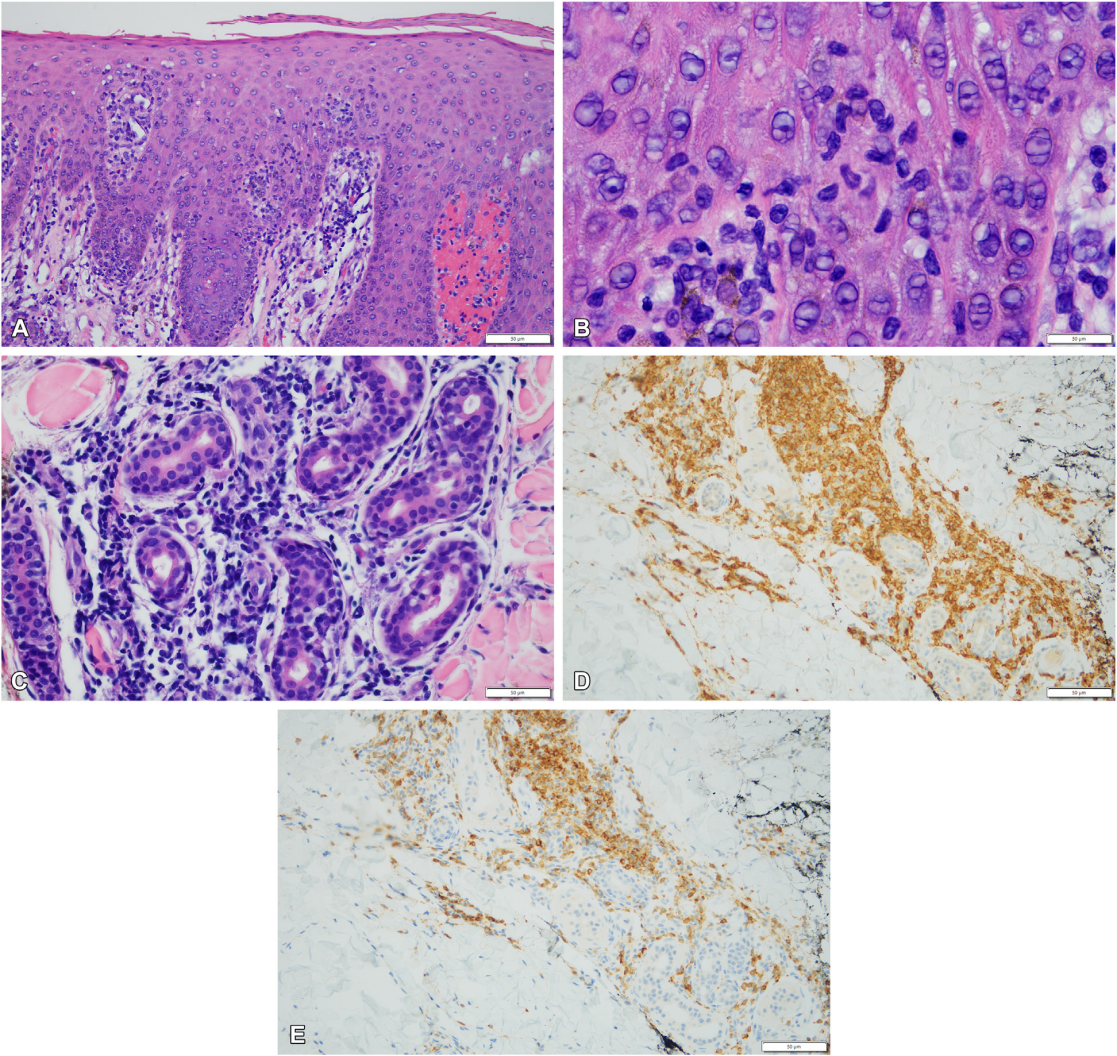
**A,** The biopsy shows an atypical epidermoptropic lymphocytic infiltrate in association with an irregular pattern of psoriasiform epidermal hyperplasia; it is a relatively passive pattern of migration into the epidermis unassociated with significant destructive interface change (100×, hematoxylin and eosin [H&E]). **B,** The lymphocytes assume a passive pattern of migration into the epidermis and are represented by small to intermediate sized lymphocytes with nuclear hyperchromasia and nuclear contour irregularity albeit without the degree of lymphoid atypia conclusive for mycosis fungoides (1000×, H&E). **C,** There is a supervening atypical syringotropic lymphocytic infiltrate surrounding and permeating the ducts and glands of the eccrine coil (400×, H&E). **D,** The extensive degree of T cell infiltration within and around the eccrine coil is highlighted by the CD3 stain (200×, diaminobenzidine). **E,** In contrast, the CD7 preparation, another pan T cell marker, is significantly diminished and corroborates the categorization of this process as a form of epidermotropic T cell dyscrasia (200×, diaminobenzidine).

Upon initial evaluation at CUMC, physical exam was notable for erythematous scaly macules and papules throughout the scalp, face, torso, and extremities without palpable lymphadenopathy (Fig 1). The biopsy showed an atypical perivascular and epidermotropic lymphocytic infiltrate with concomitant eczematoid alterations and significant tissue eosinophilia, favoring an eczematoid T-cell dyscrasia, possibly representing eczematoid MF. The hematoxylin and eosin (H&E) slides showed a haphazard epidermotropic infiltrate of atypical lymphocytes including cells with a cerebriform cytology associated with a syringotropic component including frank permeation of ducts and glands by lymphocytes. Immunohistochemistry showed a dichotomous pattern of CD4/CD8 with CD4 predominance in the dermis and CD8 predominance in the epidermis. There was a subtle reduction in CD7 amidst the atypical syringotropic lymphocytes (Fig 2).

At his follow-up evaluation at CUMC 3 weeks later, the patient had persistent pruritus with erythematous patches covering over 50% total body surface area (TBSA). Flow cytometry was negative for blood involvement and no immunophenotypic aberrancies were noted. Although a small T-cell population was detected, T-cell clonality studies were negative for a clonal population. Given the eczematoid features without clear evidence of MF, dupilumab was initiated.

The patient’s rash progressed with diffuse scaling and thickened erythroderma covering nearly 100% TBSA including skin folds without any evidence of the classic deck-chair sign (Fig 1). Two additional biopsies were consistent with an eczematoid T-cell dyscrasia and dupilumab was discontinued. Four months after initially presenting to CUMC, absolute eosinophil count was elevated to 5,509 cells/uL (reference range:15-500 cells/uL) and serum IgE was elevated to 3816 kU/L (reference: <114 kU/L) prompting referral to the emergency department. Bone marrow biopsy showed erythroid hematopoiesis with adequate progressive maturation and variable eosinophilia consistent with hypereosinophilic syndrome (HES) and negative for eosinophilic leukemia. Imatinib was started though discontinued 1 week later given the absence of neoplastic HES.

PET-CT showed hypermetabolic retroperitoneal lymphadenopathy extending along the left para-aortic region into the pelvis. Lymph node biopsy showed metastatic adenocarcinoma with primary prostate cancer. He was started on treatment with androgen deprivation therapy and an androgen receptor signaling inhibitor 6 months after his initial presentation to CUMC dermatology for his Stage IV prostate cancer. At his next follow-up appointment 1 month later, he had significant improvement in pruritus and erythroderma (Fig 1).

## Discussion

We present a case of diffuse erythroderma with biopsy proven features of an eczematoid T-cell dyscrasia in the setting of underlying metastatic prostate cancer. We propose that the patient has a unique form of paraneoplastic PEO, which we classify as a subtype of paraneoplastic erythroderma. The case also emphasizes that the absence of the deck-chair sign should not exclude PEO.

**Fig 3:**
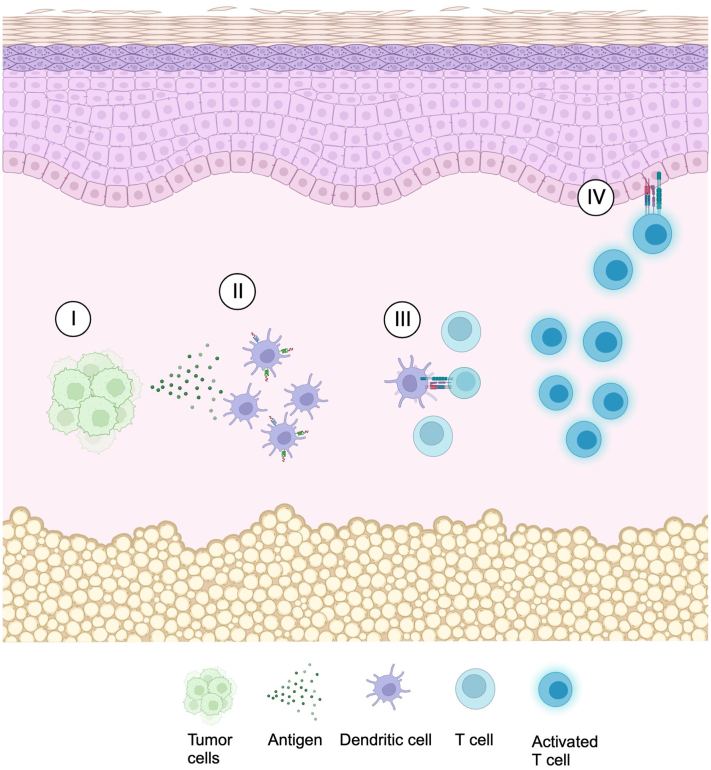
Proposed mechanism of eczematous and interface changes in paraneoplastic PEO. I: Antigen shedding by the solid tumor; II: Antigen presentation by antigen presenting cell (APC) such as a dendritic cell; III: APC activates the T cell; and IV: activated T cell cross reacts with keratinocyte antigen leading to exuberant eczematous and interface changes. *PEO*, Papuloerythroderma of Ofuji.

PEO is a rare dermatosis predominantly affecting older men with many cases originating in Japan.^1^ Typically, these patients present with pruritic papules coalescing to form erythroderma, characteristically sparing the face and skin folds (ie, deck-chair sign). Laboratory findings include eosinophilia (85%), increased serum IgE (50%), and lymphopenia (29%).^1^ Histologically, PEO often shows a pattern that is reflective of a TH2 enriched microenvironment and therefore characteristic findings are an eosinophil enriched subacute eczematous dermatitis.^1^^,^^2^ However, lymphocytic atypia is well established in PEO, most often in patients with PEO in the setting of SS/MF where histologic and phenotypic studies are compatible with cutaneous T-cell lymphoma (CTCL).^1^^,^^3^ There are cases of reversible erythrodermic T-cell dyscrasia albeit not specifically PEO usually triggered by immune dysregulatory drug therapy.^5^ The drug may function as an antigen but also dysregulates immune function to recapitulate an endogenous T-cell dyscrasia microenvironment exemplified by anticonvulsant hypersensitivity syndrome.^6^ Additionally, antigen from the solid tumor may potentially cross react with keratinocyte antigen leading to eczematous and interface changes within the epidermis reflective of a form of cutaneous systemic type IV hypersensitivity (Fig 3), similar to the proposed pathogenesis of Bazex syndrome.^7^ Due to endogenous immune dysregulation related to advanced malignancy, an overzealous immune response may have occurred resulting in a lymphomatoid expression of PEO. In fact, the excessive TH2 mediated inflammation could play a role in promoting tumor growth due to inhibition of TH1 cell-mediated immunity, providing a link between PEO and malignancy.

Moreover, lymphocytic variants of HES may also present with prominent cutaneous manifestations such as eczema, lichenification and erythroderma, similar to PEO.^8^ Lymphocytic variants of HES are classified as a subtype of reactive HES and may be identified in neoplastic conditions in which the clonal cells release factors that contribute to eosinophilic inflammation.^9^ In this case, the lack of T-cell clonality and immunophenotypic changes further suggest that this patient’s presentation is compatible with a lymphomatoid form of PEO.

Most cases of PEO have no association with disease.^3^^,^^4^ However, Ofuji proposed that multiple causative factors may underscore the development of PEO, including malignant lymphoma, hepatitis C infection, HIV/AIDS and visceral malignancy.^10^^,^^11^ Associations between PEO and visceral malignancies are particularly notable, suggesting a paraneoplastic phenomenon at least in a subset of cases. The most common visceral malignancy identified is gastric cancer, which comprises over 60% of paraneoplastic PEO cases (Table I).^2^^,^^11^, ^12^, ^13^, ^14^, ^15^

**Table I:** Reported cases of PEO-associated malignancy categorized by tumor type, including this case.

Visceral malignancy	Number of reported cases
Gastric	16
Colon	3
Prostate	3
Lung	1
Laryngeal	1
Renal	1
Hepatocellular carcinoma (HCC)	1

Gastric cancer represents the most common malignancy associated with PEO.

Given the eczematoid features on biopsy without clear features of CTCL, the patient was started on dupilumab. Dupilumab can cause a reversible and benign lymphoid reaction (LR) that mimics CTCL in eczema patients, although the exact pathophysiologic mechanism underlying this process is unknown. Clinically, both early-stage CTCL and dupilumab-induced LR commonly present with pruritic erythematous maculopapular lesions. These two conditions may also resemble each other histologically, with both often showing eczematous features such as acanthosis, parakeratosis and spongiosis, in addition to a lymphoid infiltrate. However, histologic characteristics that may distinguish MF from LR include pan-T-cell antigen loss. Additionally, atypical small/medium cerebriform lymphocytes at the dermoepidermal junction is usually present in MF whereas small cerebriform lymphocytes are scattered in the upper epidermis in LR. CD30 expression, while present in other, less common subtypes of CTCL, is unusual in early stage MF but is often identified in LR.^16^

Dupilumab has also been reported to unmask CTCL.^16^ One study even found that dupilumab can provide temporary symptomatic relief when used short-term but may be associated with progression of CTCL with long-term use.^17^ In patients with LR, discontinuation of dupilumab leads to clinical improvement of symptoms with posttreatment biopsies showing resolution of the LR with negative CD30 expression.^16^ In this case, the patient flared after treatment with dupilumab. Although dupilumab was discontinued promptly following clinical progression, the patient did not experience symptomatic relief until starting treatment for his metastatic prostate adenocarcinoma, supporting a paraneoplastic link to PEO. Moreover, biopsies taken during dupilumab treatment were consistent with biopsies prior to dupilumab, showing that it is unlikely that dupilumab unmasked an underlying CTCL in this patient.

Only 2 cases of paraneoplastic PEO related to prostate cancer have been reported which resolved with treatment of malignancy.^14^^,^^15^ Additionally, 2 cases of paraneoplastic erythroderma exist in association with prostate cancer. ^18^^,^^19^ This case contributes to the current literature on the presentation of paraneoplastic PEO especially as it pertains to prostate cancer and further suggests that treatment of the underlying malignancy may lead to clinical improvement, if not complete regression, of the erythroderma. It highlights the importance of considering paraneoplastic PEO of solid organ malignancy when biopsies show atypical lymphomatoid features that are not conclusive for CTCL and where a drug-based trigger has been excluded.

## Conflicts of interest

LJG has served as an investigator for and/or received research support from Helsinn Group, J&J, Mallinckrodt, Kyowa Kirin, Soligenix, Innate, Merck, BMS, and Stratpharma; on the speakers’ bureau for Helsinn Group and J&J; and on the scientific advisory board for Helsinn Group, J&J, Mallinckrodt, Sanofi, Regeneron, and Kyowa Kirin. OA, MHT, ERG, BAL, CMS, LMF, CC and CMM have no conflicts of interest to declare.
